# SLAM Associated Protein Signaling in T Cells: Tilting the Balance Toward Autoimmunity

**DOI:** 10.3389/fimmu.2021.654839

**Published:** 2021-04-16

**Authors:** Yevgeniya Gartshteyn, Anca D. Askanase, Adam Mor

**Affiliations:** ^1^ Division of Rheumatology, Department of Medicine, Columbia University Irving Medical Center, New York, NY, United States; ^2^ Columbia Center for Translational Immunology, Columbia University Irving Medical Center, New York, NY, United States

**Keywords:** SLAM associated protein, SAP, SH2D1A, X-linked lymphoproliferative disease, immunodeficiency, autoimmunity

## Abstract

T cell activation is the result of the integration of signals across the T cell receptor and adjacent co-receptors. The signaling lymphocyte activation molecules (SLAM) family are transmembrane co-receptors that modulate antigen driven T cell responses. Signal transduction downstream of the SLAM receptor is mediated by the adaptor protein SLAM Associated Protein (SAP), a small intracellular protein with a single SH2 binding domain that can recruit tyrosine kinases as well as shield phosphorylated sites from dephosphorylation. Balanced SLAM-SAP signaling within T cells is required for healthy immunity, with deficiency or overexpression prompting autoimmune diseases. Better understanding of the molecular pathways involved in the intracellular signaling downstream of SLAM could provide treatment targets for these autoimmune diseases.

## Introduction

T cell activation is initiated by engagement of the T cell receptor (TCR) complex and requires co-receptor signaling. While CD28 is the best characterized TCR stimulatory co-receptor, and programmed cell death protein 1 (PD-1) is the most targetable TCR inhibitory co-receptor, additional transmembrane co-receptors are responsible for the fine-tuning of antigen driven T cell responses. One family of such co-receptors is the signaling lymphocyte activation molecules (the SLAM family). These receptors, discovered approximately 20 years ago, are transmembrane proteins of the immunoglobulin (Ig) superfamily and participate in the co-activation of T cells and other hematopoietic cells (1). Following receptor ligation, intracellular signal transduction occurs by means of recruitment of the adaptor proteins SLAM associated protein (SAP) and Ewing’s sarcoma associated transcript 2 (EAT-2). SAP (encoded by the gene *SH2D1A*), is mainly expressed in T and NK cells, while it’s analog protein, EAT-2, is mainly expressed in B cells and NK cells (2, 3). Numerous recent multi-omics studies have uncovered new aspects of the biology of the SLAM family receptors and their associated adaptor proteins. This body of knowledge resulted in better understanding of the biological processes underlying adaptive immune responses, translating into novel anti-cancer therapies (4). The current publication will review the biology of these proteins, discuss novel findings implicating both SLAM and SAP in the homeostasis of T cell responses following TCR activation, and summarize published work highlighting the role of SAP signaling in the pathogenesis of autoimmune conditions.

## Mutations in *SH2D1A* Lead to X-Linked Lymphoproliferative Disease

The SAP gene (*SH2D1A*) was first cloned from patients with IgA nephropathy in 1995, however its function was initially unknown and the work was not published until years later (5). In 1998, SH2D1A was discovered to be the gene mutated in X-linked lymphoproliferative disease (XLP). *SH2D1A* is located on the long arm of human X chromosome (*Xq25*). Both loss of function mutations and deletions in the SAP gene were identified in patients with XLP (6–8). XLP affects males and is characterized by mild immunodeficiency in early life. After Epstein Barr virus (EBV) infection, patients with XLP develop uncontrolled polyclonal T and B cells proliferation, manifesting as fulminant infectious mononucleosis with hemophagocytic lymphohistiocytosis (HLH) syndrome and inability to clear the EBV infection. A hallmark feature of this condition is defective antigen specific humoral response with decreased memory B cell formation, accompanied by low serum IgG levels, ineffective immunoglobulin class switching to IgG in response to infection (9, 10), and a higher risk of developing malignant B cell lymphoma in the context of impaired immunosurveillance of B cell proliferation and education by T cells (11). Pathogenesis is attributed to a defect in SAP signaling in NK cells, resulting in profound functional defects in an anti-viral response, as well as T cells, which skews the cells’ differentiation towards a high type 1 T helper (T_H_1) IFN-y response with impaired type 2 T helper (T_H_2) and T follicular helper (T_FH_) signaling, contributing to unstable T cell - B cell interactions (12–14). The ultimate aberrant polyclonal lymphoproliferative disease results in the clinical development of hepatic necrosis, bone marrow failure, and high risk of mortality.

## SAP Is an Effector of SLAM Signaling

**Figure 1:**
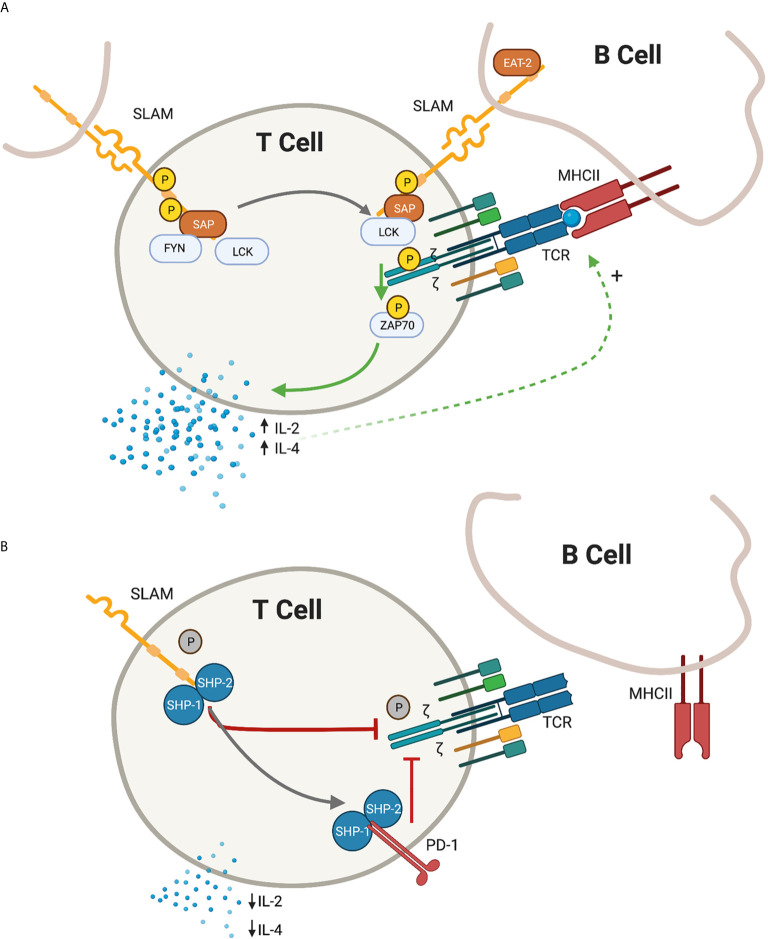
Depiction of proximal T-cell signaling following ligation of the signaling lymphocyte activation molecules (SLAM) family receptors. **(A)** In the presence of SLAM associated protein (SAP), SLAM ligation is followed by recruitment of lymphocyte specific protein tyrosine kinase (LCK) and proto oncogene tyrosine protein kinase (FYN) resulting in the phosphorylation of the cytoplasmic tail of the SLAM receptor. In the immunological synapse, SAP-LCK physically bridge the signal from the SLAM co-receptor with the antigen-specific T-cell receptor (TCR) signal, promoting phosphorylation of the zeta chain and recruitment of zeta chain of T cell receptor associated protein kinase 70 (ZAP-70), resulting in a net positive activation signal. Release of IL-2 and IL-4 further promote the antigen-specific T cell - B Cell development. **(B)** In the absence of SAP, the SLAM receptors recruit Src homology region 2 domain-containing phosphatase -1 and -2 (SHP1 and SHP2), which translate an inhibitory signal to the immunological synapse. An additional inhibitory signal from the programmed death 1 (PD-1) receptor is also enhanced in the absence of SAP signalling. IL-2 and IL-4 are relatively decreased and humoral immunity is impaired. *Green arrow, activating signal; red arrow, inhibitory signal; black arrow, transitional state.*

The SLAM family of receptors, consisting of nine members (SLAMF1/CD150 through SLAMF9), are type I transmembrane glycoproteins that are present on most hematopoietic cells. The extracellular domains act as self-ligands and can interact either in *cis* or in *trans* across cell membranes, regulating downstream signaling. In activated T cells, the SLAM receptors co-localize with the TCR complex in the immunological synapse. Following receptor cross-linking, tyrosine phosphorylation of the cytoplasmic SLAM tails initiates signal transduction cascades. This downstream signal transduction is mediated by the recruitment of the SAP adaptor molecules (**Figure 1**).

The *SH2D1A* gene that encodes SAP is 2,530 base pairs (bp) long and contains an open reading frame of 462 bp (6). The SAP protein is a 15kD and 128 amino acid cytosolic protein that consists of a single Src homology 2 (SH2) domain (98 amino acids), flanked by 5 amino acids at the N-terminus and 25 amino acids at the C-terminus (6). The presence of the Src homology 2 (SH2) domain in the small SAP molecule has suggested early on that the main function of SAP is in cell signal transduction through binding to phosphorylated tyrosines. Specifically, the cytoplasmic tails of most SLAM receptors (with the exception of SLAMF2/CD48) contain tyrosine residues that are surrounded by consensus SH2 domain binding sequences. These act as docking sites for kinases, phosphatases, and other adaptor proteins, which express a homologous SH2 binding domain. In T and NK cells, SAP binds with its SH2 domain to both phosphorylated and unphosphorylated SLAM, further attracting kinases and preventing the dephosphorylation of the SLAM receptor sites (15).

Several likely coincident and complementary mechanisms have been proposed for the role of SAP in T cell signaling. The cytoplasmic tails of the SLAM family receptors (with the exception of SLAMF2/CD48) contain between 1 (SLAMF7/CD319, mouse CRACC) and 4 (SLAMF4/CD244) immune tyrosine switch motif (ITSM) domains that serve as docking sites for SAP. Following receptor ligation, these ITSM domains are phosphorylated by protein tyrosine kinases (PTK) such as lymphocyte specific protein tyrosine kinase (LCK) and proto oncogene tyrosine protein kinase (FYN). SAP, by binding to the phosphorylated tyrosine domains of SLAM with high affinity, sterically interferes with the binding of SH2 domain-containing phosphatases, including SHIP-1, SHP1 and SHP2. In this so-called *“shielding model”* SAP prevents SLAM receptors dephosphorylation and suppression of downstream signaling (15, 16). SAP has a similar *“shielding”* function in other T cell signaling pathway. Our group recently described that SAP interacts indirectly with the PD-1 signaling complex and shields downstream phosphorylated substrates (i.e., CD28) from dephosphorylation by SHP2 (17).

In addition to displacing phosphatases, SAP potentiates the early phosphorylation events following SLAM receptor activation by directly recruiting the protein tyrosine kinases, LCK and FYN, to the cytoplasmic tail of SLAM (18, 19). SAP has two distinct binding sites: it can interact with phosphotyrosine domains *via* residues surrounding arginine 32 of SAP, or with SH3 domains *via* residues surrounding arginine 78 of SAP (8, 20, 21). Specifically, SAP can bind both the SH3 domain as well as, weakly, the kinase domain of FYN; yet it interacts solely with the kinase domain of LCK (20, 22). Furthermore, SAP can simultaneously interact with FYN’s SH3 domain and with the SLAM receptor’s phosphotyrosine site, forming a trimolecular complex (20, 21). In this manner, acting as a bridge between the FYN kinase and the SLAM receptor, SAP has an important role in promoting downstream phosphorylation events in the signaling cascade (20, 21).

The interaction of FYN with the SLAM-SAP complex is dependent on the engagement of SLAM by extracellular ligands, which triggers a conformational change in the SLAM-SAP complex, allowing for FYN binding (23). Recruitment of FYN promotes the phosphorylation of SLAM itself, as well as other downstream intermediates including inositol phosphatase SHIP-1 and adaptor Dok-related molecules (18). At other times, it is the recruitment of LCK rather than FYN that has been observed in association with SLAM-SAP signaling (24). LCK plays a critical role in initiating the signaling cascade following TCR activation by phosphorylating the zeta chains of the TCR, leading to the binding and activation of the kinase ZAP70. The ability of SAP-LCK to interact both with the SLAM receptors as well as directly with the CD3 zeta chain, suggests that SAP has a role in physically bridging co-receptors, as well as bringing kinases such as LCK to the immunological synapse, promoting downstream signaling cascade (25). This is further supported by the finding that in activated T cells, SAP interacts with SLAMF6/CD352 (human NTB-A/mouse Ly108) and is associated with the activation of the ERK and AKT pathways downstream of ZAP70 (25, 26). Furthermore, in the absence of SAP, signaling through SLAM receptors results in increased recruitment of SHP1 phosphatase. Targets of SHP1 include the SRC protein kinases, such that SAP deficiency is associated with reduced phosphorylation of these kinases at the immunologic synapse (27, 28), suggesting that an alternative, negative, intracellular signal pathway predominates in the absence of normal SLAM-SAP interaction. Indeed, SLAMF6-SHP-1 interaction, enhanced in the absence of SAP, associate with and promote SHP-1-mediated TCR zeta dephosphorylation, reducing proximal TCR signaling. (Chu et al. JI) In summary, SAP, as an adaptor molecule, has at least two complementary mechanisms of action to potentiate SLAM signaling: *1)* SAP sterically *“caps”* the ITSM motifs on the cytoplasmic tail of the transmembrane SLAM, preventing phosphatase binding and dephosphorylation, and *2)* SAP directly recruits PTKs to the SLAM receptor site, further promoting phosphorylation not just of the SLAM receptors, but also the TCR zeta chain, thereby bridging the SLAM co-receptor with the TCR and enhancing the downstream activation signal.

## SAP Signaling Downstream of SLAMF6 Supports Germinal Center Formation

SAP deficiency is associated with defective B cell proliferation, impaired germinal center formation and dysgammaglobulinemia. Indeed, mice lacking SAP show a near complete absence of virus-specific long-lived plasma cells and memory B cells. Interestingly, this defect is extrinsic to B cell signaling and is the result of the aberrant CD4 T cell functions (29). Using intravital two-photon microscopy, it was shown that SAP deficiency selectively impairs the ability of CD4 T cells to stably interact with cognate B cells, resulting in inadequate levels of contact-dependent T cell education. Consequentially, there is an aberrancy in the development of the germinal centers and proliferation of antigen specific B cells (13). Early work implicated a skewed T_H_1 *vs.* T_H_2 CD4 T cell development as a result of decreased SAP/FYN signaling, with decreased IL-4 cytokine production leading to abnormal B cell development (30–32). Subsequent work identified an essential requirement for SAP in the development of T_FH_ cells in the germinal center, absence of which is associated with decreased T_FH_ IL-4 production, loss of T_FH_-B cell help activity, and decreased antibody production (33–35). Additionally, a cytokine independent, T-cell intrinsic defect contributes to the impaired B cell development such that SAP deficient CD4 T cells exhibit decreased and delayed inducible co-stimulator (ICOS) expression and aberrant CD40L expression (36).

As mentioned, it is the expression of SAP in T cells, and not in B cells, that is required and sufficient for SAP-dependent antibody production and germinal center formation (37). Specifically, it is the homophilic interaction of the T cell - B cell immunological synapse that has an essential role in B cell development. B cells co-cultured with either SAP deficient or SLAMF6 (human NTB-A/mouse Ly108) deficient CD4 T cells have decreased B cell survival (38). On the other hand, while isolated SAP deficiency is associated with impaired germinal center formation, the deletion of SLAMF6 (human NTB-A/mouse Ly108) in SAP deficient CD4 T cells rescues normal germinal center formation (28). In the absence of SAP, the negative signal from SLAMF6 (human NTB-A/mouse Ly108) to the CD4 T cell is mediated by recruitment of SHP-1 to the T cell - B cell synapse (39). Thus, SLAMF6 (human NTB-A/mouse Ly108) can transmit positive (via SAP) and negative (*e.g., via* SHP-1) signals to the immunological synapse, determining the fate of B cell development and humoral immunity (27). Indeed, the aberrant humoral response in SAP deficiency can be rescued by reconstitution with SAP-mutated T-cells that exhibit decreased SAP-FYN binding; suggesting a Fyn independent function of SAP either by means of recruiting to SLAM other kinases (such as LCK) or by displacing the negative (SHP-1/SHP-2) phosphatases (36).

In contrast to the unstable B cell interaction, SAP deficient T cells are still able to form stable conjugates with dendritic cells (13), suggesting that impaired SLAM-SAP signaling predominantly affects T cell - B cell interactions.

In summary, balanced positive vs. negative signals transmitted from the SLAM receptors to the TCR, modulated by the presence and absence of SAP respectively, is essential for stable T cell - B cell interactions, development of T_H_2 and T_FH_ T cell subsets, formation germinal centers and proliferation of antigen specific B cells.

## SAP and SLAMF6 Play a Role in the Homeostasis of T Cell Responses

Homeostatic mechanisms which curb excess T cell activation following an acute immune response rely on the self-regulatory clearance of T cells following TCR re-stimulation, known as re-stimulation-induced cell death (RICD) (40, 41). An impaired RICD response was described in ovalbumin peptide-activated SAP deficient T cells as compared with their wild type counterparts (42). The association between SAP deficiency and a failing apoptotic response was confirmed also in patients with XLP patients, where the impaired contraction of the immune response following initial activation results in hyperproliferation of polyclonal T cells and severe systemic inflammation. Introduction of SAP expression into the lymphoblastoid cells lines established from XLP patients rescued the apoptotic response (43). Thus, a pro-apoptotic function of SAP exists by which it contributes to the maintenance of T cell homeostasis, whereas failure of this mechanism may be especially relevant in conditions of chronic stimulation, such as infection, cancer or autoimmunity.

A strong antigen-driven TCR response, with adequate IL-2 signaling, is required for RICD (44). In a SAP dependent manner, SLAMF6 in human T cells associates with LCK, but not with FYN, to amplify proximal TCR signaling in RICD response (24). On the other hand, FYN is preferentially recruited by SAP in T cells with aberrant SLAMF6-SAP signaling, decreased IL-2 production, and resistance to RICD (45). Thus, SAP-SLAMF6 signaling, mediated by differential kinases recruitment and association with TCR, regulates the apoptotic response following T cell activation. Interestingly, FOXP3 regulatory T cells (Treg) express very low levels of SAP and are resistant to RICD, compared to conventional T cells. The transcription factor FOXP3 directly binds to and represses *SH2D1A* promoter. Indeed, ectopic SAP expression restores RICD sensitivity in human FOXP3^+^ Treg, highlighting the essential role of SAP in the T cell apoptosis response (46).

Further linking SAP signaling to the function of contracting T cell activation and maintaining homeostatic following activation, our group has recently reported a novel association of SAP with the T cell checkpoint inhibitor PD-1. Specifically, the silencing of SAP in primary human T cells enhanced PD-1 ligation and function, resulting in the inhibition of IL-2 secretion. Furthermore, T cells from patients with XLP, who lack functional SAP, were hyper-responsive to PD-1 signaling. Complementary, overexpression of SAP abrogated the inhibitory effect of PD-1 (17, 47). Thus, while RICD is impaired in SAP deficiency, PD-1 signaling is enhanced, possibly in a compensatory attempt to maintain T cell signal regulation and balance. Future work confirming and advancing these initial findings is needed to better understand the complex interplay of SLAM/SAP and PD-1 on TCR activation.

## SAP and SLAMF6 Contribute to the Pathogenesis of Autoimmunity

Given the important function of SLAM and SAP signaling in T cell differentiation and humoral immunity, it is not surprising that the aberrant signaling pathways of these molecules have been implicated in the pathogenesis of autoimmunity. Systemic lupus erythematosus (SLE) is a prototypic autoimmune condition characterized by an overly active humoral immune response with a loss of self-tolerance and autoantibody production, leading to systemic inflammation and organ damage. Mice deficient in SAP are resistant to experimental models of lupus (pristane-induced SLE and Murphy Roths Large (MRL)/lymphoproliferation (lpr) spontaneous SLE models), and this protection is attributed to impaired development of humoral immunity associated with defective germinal center formation and subsequently decreased antibody, including autoantibody, response (48, 49). SAP expression in T cells, but not in B cells, was essential to restore the development of autoimmune arthritis, echoing the findings from XLP patients where SAP dependent T cell differentiation was critical for developing a humoral response (50, 51). On the other hand, similar to the pathological T cell signaling in XLP, SAP deficient mice had increased IFN-y production and enhanced susceptibility to experimental autoimmune encephalomyelitis (EAE, the mouse model of multiple sclerosis), possibly as a result of skewing the immune system to a Th1 mediated autoimmunity (48). At the same time, SAP deficiency in XLP patients is associated with loss of self-tolerance in B-cells and increased autoantibody (HEp-2 reactive antibody) formation (52). Similarly, conflicting reports of SAP expression in SLE exist. In one study, CD4 T cells from SLE patients, as compared to healthy control CD4 T cells, had increased SAP protein expression (53). Similarly, a human susceptibility locus for SLE in the *SH2D1A* gene itself was associated with increased gene activity as compared to the reference allele (54). On the other hand, SAP levels were reported to be decreased in T cells from SLE patients, and increased degradation of SAP by caspase 3 was cited as at least one of the contributing mechanisms (55). Given single observation reports and significant variability in disease activity and baseline medication use among the SLE patients, these findings need to be validated in future works. Still, as with many other signaling proteins, it is likely that both overexpression and deficiency in SAP signaling can drive autoimmunity, with either extreme tipping the delicate balance of adaptive immunity.

Genome wide association studies in human SLE and in spontaneous murine lupus models of SLE have identified the *1q23* region on chromosome 1 as a susceptibility locus for SLE (56). Subsequently identified as the *SLE1* locus, these functionally related genes first identified in congenic mouse strains carrying the NZM susceptibility interval on a C57BL6 background, mediate loss of tolerance to nuclear antigens and contribute to SLE pathogenesis [Morel 2001]. This *SLE1* locus codes for the entire family of SLAM molecules; with polymorphisms contributing to the loss of self-tolerance, autoantibody production and susceptibility to spontaneous development of SLE (57–59). It was subsequently discovered that the locus encoding for Ly108, the murine equivalent of SLAMF6/CD352 (human NTB-A/mouse Ly108), had the strongest association with the disease. Specifically, two dominant alternatively spliced isoforms were identified in lymphocytes: Ly108-1 in the lupus-prone mice and Ly 108-2 in the non-lupus prone mice. Ly108-1 was associated with a modified tyrosine-based motif that promoted enhanced SAP-mediated Fyn recruitment, resulting in enhanced tyrosine phosphorylation signaling in T cells, loss of tolerance, and increased survival in B cells (58–60). On the other hand, subsequent discovery of a third splice variant isoform of the Ly108 protein, Ly108-H1, ameliorates development of lupus in mouse transfer models of SLE (61). Unlike the enhanced phosphorylation seen in the Ly108.1 lupus-prone splice variant, Ly108-H1 is resistant to tyrosine phosphorylation and may act as a decoy isoform mitigating total Ly108 signaling and thereby conferring protection from lupus autoimmunity (62). Thus, different splice variants of the Ly108 gene may predispose or protect from SLE by modulating intracellular SAP recruitment and SLAMF6/Ly108 receptor phosphorylation, affecting strength of TCR signaling and ultimately B-cell development and autoantibody production.

Mixed reports of SLAMF6/Ly108 expression and function in autoimmunity exist. Increased T cell SLAMF6 expression in SLE was described in earlier work (63) but more recent work found no difference in SLAMF6 expression between SLE and healthy controls (64, 65). Confounding these results is the challenge of controlling for immunosuppressive medication use, which has been shown to affect SLAM surface expression in patients with rheumatoid arthritis (66). Cross-linking of SLAMF6 and CD3 in healthy human T cells activates the GTPases Ras and Rap 1, leading to T cell proliferation with enhanced IFN-y production and Th1 differentiation, with a reciprocally reduced IL-4 secretion and impaired Th2 differentiation (67, 68). On the other hand, others have reported that deficient SLAMF6/Ly108 signaling reduces BCR signaling, lowers the frequency of B-T cell conjugates, resulting in loss of self-tolerance, culminating in autoantibody production and autoimmunity (69). Once again, it appears that a healthy balance of SLAM-SAP signaling is required to maintain functional immunity, with both extremes predisposing to autoimmunity. Importantly, the ultimate function of any SLAM receptor cross-linking may depend on the delicate balance of the intracellular signal cascade proteins, such as the binding SLAM by SAP *vs.* SHPs or the recruitment of LCK *vs.* FYN, to determine the eventual effect on TCR signaling and the fate of the adaptive immune response (24, 28, 45).

In summary, SLAMF6-SAP signaling promotes humoral immunity and contributes to SLE risk whereas knockout models of SAP are protective against SLE. Instead, SAP deficiency skews towards Th1 differentiation and a high IFN-y state, thereby swinging the pendulum and increasing risk of T cell mediated autoimmunity.

## Other SLAM Family Receptors’ Contribution to Autoimmunity Risk

Other members of the SLAM family have also been implicated in the pathogenesis of autoimmune disease. An SLE susceptibility locus in the *SLAMF3/CD229* (mouse Ly9) gene results in a single amino acid change, localized in the SH2 binding domain region; leading to stronger association of SLAMF3 with SAP and increased T cell activation following TCR stimulation (70, 71). SLAMF3/CD229 expression is increased on the surface of SLE T cells compared with normal cells. SLAMF3 signaling promotes T cell proliferation and Th17 differentiation with upregulation of IL-17 synthesis, a critical driver of SLE disease activity (63, 72, 73).

Meanwhile, polymorphisms in the *SLAMF4/CD48 (mouse 2B4)* gene have been described to be associated with rheumatoid arthritis risk (74). There is also evidence that SLAMF4/CD48 expression, a receptor associated with cytolytic activity and granzyme release, is reduced in SLE T cells (75, 76). More recently, a novel CD4 T peripheral helper (Tph) cell population, PD-1^hi^CXCR5^-^CD4^+^, was found to be expanded in rheumatoid arthritis (77). These cells, thought to infiltrate inflamed tissue and provide B cell helper function in an ectopic lymphoid tissue setting, express high levels of SAP as well as SLAMF1/CD150, SLAMF5/CD84 and SLAMF6/CD352 (human NTB-A/mouse Ly108). Furthermore, while Tph cells co-cultured with B cells induce plasma cell differentiation, antibody blockade of SLAMF5, but not SLAMF6, is sufficient to block plasma cell differentiation and IgG production in these assays.

SLAMF7/CD319 (mouse CRACC) has also been implicated in autoimmunity, although with mixed findings. SLAMF7 expression is decreased on SLE CD8 T cells, but is increased on IFN-α expressing plasmacytoid dendritic cells as well as SLE B cells and plasmablasts compared to healthy control B cells (64, 75, 78, 79). Anti-SLAMF7 antibody, aimed at targeting antibody producing plasma cells, reduced autoantibody formation and disease severity in mouse models of collagen induced arthritis (80). More recently, SLAMF7/CD319 was also found to be differentially upregulated on monocytes from inflamed rheumatoid arthritis synovial tissue, as compared to osteoarthritis synovium; SLAMF7 signaling on these monocytes induced super-activation and inflammatory cytokine release (81).

Finally, while our review is focused on T cell - B cell signaling, the SLAM receptors are expressed on most hematopoietic cells, including early progenitor cells, myeloid cells, and lymphoid cells, and all these likely contribute to the overall immune response. In the thymus, the SLAM/SAP signaling pathway is critical for the development of unconventional, non-MHC restricted, IL-17 and IFN-γ producing γδ T cells that likely play a unique and important role in autoimmunity (82, 83). In phagocytic myeloid cells, the SLAM receptors contribute to microbial pathogen recognition and regulation of cell trafficking and microbicidal activity in the inflammatory state (84). In the lymphoid lineage, engagement of the SLAM receptors on the NK cells similarly induces tyrosine phosphorylation and recruitment of the SAP-family adaptors, potentiating NK cell cytotoxicity and cytokine production (85). However, while dysregulation of NK cell function has been described in autoimmunity, the role of NK cell signaling in the pathogenesis of the disease remains incompletely understood and is reviewed elsewhere (86).

## Conclusions

In summary, SAP is a small protein that associates with the cytoplasmic tail of the SLAM family receptors in T and NK cells and functions in signal transduction, drawing kinases to the cell membrane and bridging the signal across to the TCR site. The majority of the evidence suggests that SAP promotes T cell activation, although it also has a role in contracting the T cell response following activation. SAP is essential for the formation of stable T cell - B cell interactions in mounting an antigen-specific B cell response and immunoglobulin production. SLAMF and SAP polymorphisms confer susceptibility to autoimmunity. SAP deficiency is associated with T_H_1-IFN-y driven pathology and SAP hyperactivity, although less well categorized, predisposes to an exaggerated humoral response and autoantibody production. Further research aimed at better understanding the function of the SLAM family receptor signaling across the cells of the innate and humoral immune system is needed to pave the way for therapeutic interventions in the future.

## Author Contributions

YG drafted the manuscript and the figure. AA and AM conceived and revised the review. All authors contributed to the article and approved the submitted version.

## Funding

This work was supported by grants from the NIH (AI125640, CA231277, AI150597), the Cancer Research Institute, and the Lisa M. Baker autoimmunity innovation fund (AM), and the National Center for Advancing Translational Sciences: Clinical and Translational Science Award TL1TR001875 (YG).

## Conflict of Interest

The authors declare that the research was conducted in the absence of any commercial or financial relationships that could be construed as a potential conflict of interest.
